# Glycyrrhizic Acid Protects Experimental Sepsis Rats against Acute Lung Injury and Inflammation

**DOI:** 10.1155/2022/3571800

**Published:** 2022-08-29

**Authors:** Jiali Shen, Zhongjie Hua, Yueyang Chai

**Affiliations:** ^1^Department of Emergency, The First People's Hospital of Pinghu, Pinghu 314200, Zhejiang, China; ^2^Department of Emergency Medicine, Second Affiliated Hospital, School of Medicine, Zhejiang University, Zhejiang 310006, China

## Abstract

**Background:**

The incidence of acute lung injury/acute respiratory distress (ALI/ARDS) is high in sepsis aggravating morbidity and mortality. Glycyrrhizic acid (GA) has pharmacological activities in the treatment of inflammation and antiviral.

**Materials and Methods:**

Sepsis rats were constructed by the cecal ligation and puncture (CLP) surgery. After GA (25 and 50 mg/kg) injection, the survival rate, blood oxygen, biochemical indexes, myeloperoxidase (MPO) activity, and wet/dry weight ratio of the lung were observed. The bronchoalveolar lavage fluid was collected to count the cells and measure the level of TNF-*α*, IL-1*β*, IL-10, and high mobility group box-1 protein (HMGB1). Lung tissue sections were taken to observe the levels of histopathological injury and apoptosis by HE and TUNEL staining. The levels of HMGB1, TLR4, p-38 MAPK, NF-*κ*B, and ERK1/2 proteins were observed by immunohistochemistry and Western blot.

**Results:**

GA treatment improved the survival rate, blood oxygen, ALT, AST, BUN, and Scr of CLP rats. It could advance the MPO activity, the wet/dry weight ratio, histopathological injury, apoptosis, and the IL-10 level in the lung. After GA injection, the number of total cells, neutrophils, and macrophages in the CLP rats was reduced and the levels of TNF-*α*, IL-1*β*, HMGB1, TLR4, p-38 MAPK, and ERK1/2 in the CLP rat were also repressed.

**Conclusions:**

GA treatment may improve the sepsis-induced ALI/ARDS and inflammation by inhibiting HMBG1. This study provided an experimental basis for the prevention and treatment of ALI/ARDS caused by sepsis.

## 1. Introduction

Sepsis is a highly inflammatory disease caused by bacteria, fungi, or viruses, which leads to organ dysfunction after infection [1, 2]. Acute respiratory distress (ARDS) is a severe form of acute lung injury (ALI) [3]. The incidence of pulmonary dysfunction and ALI/ARDS was high in sepsis aggravating morbidity and mortality [4, 5]. There was still no effective treatment for sepsis-induced ALI and it is urgently necessary to study and develop the pathogenesis and treatments of the ALI [6]. Studies found that pathogenesis involves the secretion of a series of inflammatory cytokines and the inhibition of related genes can inhibit inflammation and improve ALI [7, 8]. Cao's study suggested that reducing inflammation against LPS-induced ALI was an effective method [9]. The occurrence and development of ALI are closely related to the outbreak of inflammation [10]. High mobility group box-1 protein (HMGB1), a widely distributed and highly conserved nucleoprotein gene, is involved in activating inflammation-related pathways such as nuclear factor-*κ*B (NF-*κ*B) and Toll-like receptor4/p38 mitogen-activated protein kinase (TLR4/p-38 MAPK) [11, 12]. In glaucoma mice, researchers found that HMGB1 can participate in the promotion of inflammation through the NF-*κ*B pathway [13].

Glycyrrhizic acid (GA), a triterpene compound isolated from licorice, has pharmacological activities in the treatment of inflammation and antiviral [14–16]. GA can improve the inflammation and fibrosis mediated by HMGB1 in the pulmonary toxicity model in mice [17]. The pretreatment of GA could antagonize sepsis and improve renal injury [18]. Moreover, Chen found GA significantly inhibited lipopolysaccharide-induced ALI and reduced the realization of pro-inflammatory cytokines such as MCP-1, COX2, and HMGB1 [19]. Therefore, we suspected that GA might improve acute lung injury and inflammation in the cecal ligation and puncture (CLP) rats by regulating HMGB1. To verify the conjecture, we selected CLP model rats to observe ALI and inflammation in CLP rats by observing lung function indexes, biochemical indexes, and inflammation to observe the biological effect and mechanism of GA on sepsis-induced ALI.

## 2. Materials and Methods

### 2.1. Main Materials

Sprague-Dawley (SD) rats (200–230 g) were bought from Shanghai Jihui Laboratory Animal Care Co., Ltd, China (SCXK (Hu) 2017–0012). Glycyrrhizic acid (GA) (≥95%, S24734) was bought from Shanghai Yuanye Bio-Technology Co., Ltd., China. The HMGB1 (MM-0486R1), myeloperoxidase (MPO) (MM-0337R1), tumor necrosis factor-*α* (TNF-*α*) (MM-0132M1), interleukin-1*β* (IL-1*β*) (MM-0040M1), and interleukin-10 (IL-10) (MM-0195R1) enzyme-linked immunosorbent assay (ELISA) kits were all provided by Meimian Industrial Co., Ltd, Jiangsu, China. Hematoxylin-eosin staining (HE) kit (G1003), protease K (G1205), the cell-permeable fluid (G1204), DAPI Staining Solution (DAPI, G1012), TUNEL kit (G1501), anti-fade mounting medium (G1401), and DAB Horseradish Peroxidase Color Development Kit (G1211) were supplied by Wuhan Servicebio Technology Co., Ltd., China. The RIPA lysate buffer (P0013D) was bought from Shanghai Beyotime Biotechnology Co., Ltd., China. The anti-HMGB1 (AF7020), anti-TLR4 (AF7017), anti-NF-*κ*B p65 (AF5006), anti-extracellular signal-regulated kinase l/2 (ERK1/2) (AF0155), anti-P-ERK1/2 (AF1015), anti-p-NF-*κ*B p65 (AF2006), and anti-GAPDH (AF7021) antibody were purchased from Jiangsu Affinity Biosciences Co., Ltd. and were diluted according to the product instructions. The anti-p-38 MAPK and anti-p-p38 MAPK antibodies were bought from Abcam, Cambridge, UK. The anti-rabbit IgG (7074) was provided by CST, MA, US. The ECL chemiluminescence kit (PE0010) was bought from Beijing Solarbio Co., Ltd.

### 2.2. Model Establishment

The model of sepsis rats was established by the CLP surgery [20]. The CLP rats were anesthetized with isoflurane and then cut from the middle of the abdominal wall. The cecum was gently pulled out to ligate at the cecum's midpoint with sterile No. 4 thread. Then, a 21 G sterile needle was used to puncture the perforation 4 times in the middle between the ligature site and the top of the cecum. The rats were sutured and resuscitated with 5 mL/100 g of normal saline. Lastly, rats' pain was relieved by buprenorphine (0.05 mg/kg). The CLP rats were intraperitoneally injected with GA (Low/high dose: 25 mg/kg or 50 mg/kg) or physiological saline (5 mL/100 g) 1 h after the operation. The sham rats underwent surgery similar to CLP rats, including intraperitoneal injection with physiological saline, anesthesia, and abdominal midline incision for except cecal ligation and puncture. The survival of 10 rats in each group was observed every 24 h after the operation.

### 2.3. Animal and Grouping

The CLP group (*n* = 16): Intraperitoneal injection of physiological sodium was gone on 1 h after CLP operation. The CLP + L-GA group (*n* = 16): Intraperitoneal injection of 25 mg/kg GA at 1 h after CLP operation. The CLP + H-GA group (*n* = 16): Intraperitoneal injection of 50 mg/kg GA at 1 h after CLP operation. The sham group (*n* = 16): Intraperitoneal injection of physiological sodium was gone on 1 h after CLP operation. The Animal Experimentation Ethics Committee of Zhejiang Eyong Pharmaceutical Research and Development Center, whose license number is SYXK(Zhe) 2021–0033, reviewed and approved all experimental work for this paper. The rats have been raised on a free diet, at 60 ± 10% humidity and 20 ± 2°C temperature, and the environment was circulated day and night for 12 h.

### 2.4. Sample Acquisition

After 24 h of the experiment, the rats were anesthetized with 2% pentobarbital sodium and then underwent thoracotomy for blood [21]. Some rats were perfused with pre-cooling PBS to take the right lungs and collect bronchoalveolar lavage fluid (BALF). The wet to dry (W/D) weight of the left lung tissue was measured. The above operations were carried out by professional technicians. Blood was used to prepare serum and detect biochemical indexes. The lungs of the remaining rats were used for histopathological observation or kept at −80°C.

### 2.5. The Detection of Myeloperoxidase (MPO) and Blood-Gas Index in Lungs

The lung tissues were rinsed with physiological saline and the lung tissue homogenate was prepared and used to test the MPO according to the instructions of the MPO kit. The pulmonary blood-gas index was detected by the manual blood gas analyzer.

### 2.6. The BALF Analysis

The count of macrophages, neutrophils, and total cells in the BALF was observed by a hemocytometer. Moreover, the level of TNF-*α*, IL-1*β*, IL-10, and HMGB1 in BALF were analyzed by ELISA kits based on the protocols of the manufacturer.

### 2.7. Bacterial Culture

The blood of rats was homogenized in 2 mL sterile PBS. After 10 times gradient dilution, they were placed on the lysogeny broth agar plate for bacterial culture for 24 h at 37°C and the colonies were counted. The results are expressed in colony-forming units (CFU)/mL.

### 2.8. Histopathological Evaluation of Lungs

Lung histopathology was evaluated by HE. After dewaxing, the sections made of tissue paraffin blocks were stained with HE kits according to the product guidelines and then observed with the microscope (Nikon Eclipse C1, JPN) after sealing. The scoring criteria of lung histopathological injury include 4 aspects: the degree of alveolar hemorrhage; the degree of hyperemia; the levels of infiltration and the accumulation of neutrophils in the alveolar space and vascular wall; and the degree of pulmonary alveolar wall increment in thickness and hyaline membrane formation. It was given marks of 0–4 from light to heavy.

### 2.9. TUNEL Staining

The tissue sections were treated with dimethylbenzene and absolute alcohol gradients. Moreover, they have been covered with protease K (10%). After removing the liquid, the cell-permeable fluid was added to cover the tissue for 20 min. After cleaning, the samples were incubated in the wet box for 2 h with the TUNEL kit. Finally, it was incubated with DAPI and sealed with an anti-fade mounting medium. DAPI (blue) and positive cells (green) were observed under the fluorescence microscope. The positive cell rate was the proportion of positive cells in total cells.

### 2.10. Immunohistochemistry (IHC) Assay

After treatment with dimethylbenzene and absolute alcohol gradients, tissue paraffin was repaired with sodium citrate buffer (pH = 6). Then, 3% H_2_O_2_ and 3% BSA were used to block peroxidase activity and antigens. In sequence, the processed sections were incubated with a HMBG1 antibody and HRP-labeled rabbit secondary antibody. After the color treatment with the DAB chromogenic agent, the nuclei were counterstained with DAPI. Lastly, the slices were sealed with a glycerol jelly mounting medium. Microscopically, blue is the nucleus and brown is the positive reaction point of HMBG1.

### 2.11. Western Blot (WB)

The lung tissues of each group were lysed to extract total protein. The proteins that were mixed with loading buffer in the ratio of 1 : 4 and were denatured in boiling water for 5 min. The samples were differentiated in SDS-PAGE concentrated rubber by electrophoresis. Later, they were transferred to the polyvinylidene fluoride membrane by electroblotting. Following, 5% nonfat milk was used to block the samples. After washing, they were incubated with antibodies of HMGB1 (1 : 1000, 20 kDa), TLR4 (1 : 1000, 100 kDa), p-p38 MAPK (1 : 1000, 41 kDa), p38 MAPK (1 : 1000, 41 kDa), p-ERK1/2 (1 : 2000, 42,44 kDa), ERK1/2 (1 : 2000, 42,44 kDa), NF-*κ*B (1 : 1000, 65 kDa), p-NF-*κ*B (1 : 1000, 65 kDa), and GAPDH (1 : 10000, 37 kDa) at 4°C overnight. Then, they were washed by TBST and blocked by nonfat milk. Subsequently, the samples reacted with anti-rabbit IgG for 2 h at 25°C. Then, the film was washed again. After the film was treated with ECL chemiluminescence kits, it was imaged with a chemiluminescence instrument (610020-9Q, Qinxiang, Shanghai, CHN).

### 2.12. Statistical Analysis

Data analysis by SPSS 16.0. Between multiple groups of measurement data by One-way ANOVA, the independent sample *t* test was used between groups. All data were expressed as mean ± standard deviation ($\bar{x}$ ±*s*), *p* < 0.05 means the difference was statistically significant.

## 3. Results

### 3.1. The Effects of GA on Bacterial Infection, Survival, and Biochemical Indexes in CLP Rats

**Figure 1:**
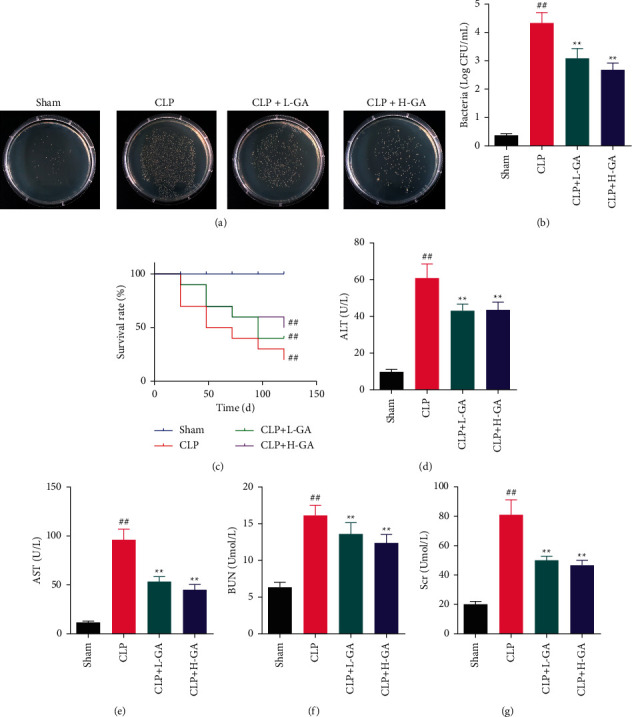
The levels of bacterial proliferation, survival, and biochemical indexes in experimental sepsis rats ($\bar{x}$ ±*s*). (a, b) The photographs and number of bacterial infections in the blood (*n* = 6). (c) The survival rate of rats at 0 h, 24 h, 48 h, 72 h, 96 h, and 120 h after cecal ligation and puncture (CLP) operation (*n* = 10). (d–g) The levels of alanine aminotransferase (ALT), aspartate aminotransferase (AST), serum creatinine (Scr), and blood urea nitrogen (BUN) (*n* = 6). GA: glycyrrhizic acid. ^##^*p* < 0.01*vs.* the Sham group; ^*∗∗*^*p* < 0.01*vs.* the CLP group.

The bacterial culture experiment was carried out on the blood of the four groups (Figure 1(a)). The number of bacteria in the CLP group was remarkably larger than that in the Sham group (*p* < 0.01), while the numbers in the CLP + L-GA group and the CLP + H-GA group were immensely lesser than that in the CLP group (*p* < 0.01).

Observing the survival of rats, it was found that the proportion of living rats in the model rats (CLP group) was 20% and the survival rate of CLP rats treated with GA was improved. The proportion of living rats in the CLP + L-GA group was 40% and in the CLP + H-GA group was 50% (Figure 1(b)).

The statistical analysis results of ALT, AST, Scr, and BUN are shown in Figure 1(c) to evaluate the degree of liver and kidney injury. The four indexes in the CLP group were enormously upper compared to the rats in the Sham group (*p* < 0.01). Moreover, in the CLP + L-GA group and the CLP + H-GA group, the four biochemical indexes were notably lesser than those in the CLP group (*p* < 0.01).

### 3.2. The Effects of GA on the MPO Activity, the Wet-to-Dry Weight Ratio of the Lung, and Blood Oxygen in CLP Rats

**Figure 2:**
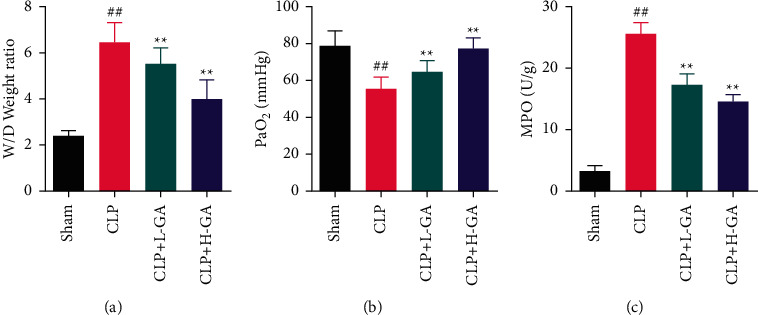
The levels of the myeloperoxidase (MPO) activity, the wet-to-dry (W/D) weight ratio of the lung, and blood oxygen in cecal ligation and puncture (CLP) rats ($\bar{x}$ ±*s*). (a) The W/D weight ratio of the lung (*n* = 6). (b) The blood oxygen in rats (*n* = 6). (c) The MPO activity in the lung (*n* = 6). GA: glycyrrhizic acid. ^##^*p* < 0.01*vs.* the Sham group; ^*∗∗*^*p* < 0.01*vs.* the CLP group.

In the activity of MPO and the W/D weight ratio of the lung, the CLP group was tremendously superior to the Sham group, but this ratio in the GA treatment rats (the CLP + L-GA and CLP + H-GA groups) was markedly reduced (*p* < 0.01). Compared with the Sham group, the blood oxygen concentration in the CLP group was notably smaller (*p* < 0.01), it in the CLP + L-GA group and CLP + H-GA group was a cut above the CLP group markedly yet (*p* < 0.01) (Figure 2).

### 3.3. The Effects of GA on Injury and Apoptosis in the Lung Tissues of CLP Rats

**Figure 3:**
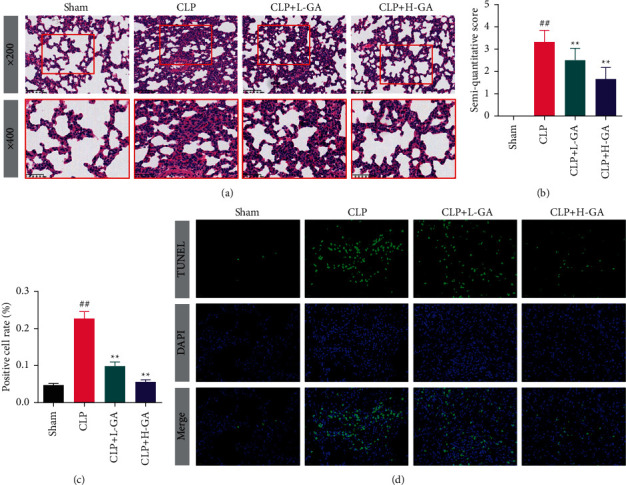
The observation of pathological injury and apoptosis of lung ($\bar{x}$ ±*s*). (a, b) The results of hematoxylin-eosin (HE) staining (*n* = 6, ×200 and ×400). (c, d) The results of TUNEL staining (*n* = 6, ×400). GA: glycyrrhizic acid, CLP: cecal ligation and puncture. ^##^*p* < 0.01*vs.* the Sham group; ^*∗∗*^*p* < 0.01*vs.* the CLP group.

The pathological injury of the lung was observed by HE (Figures 3(a) and 3(b)). In the Sham group, the structure of the lungs was normal and clear. In comparison to the Sham group, images from HE staining of the CLP group were observed with more apparent infiltration of inflammatory cells, thicker tracheal wall, and more irregular lumen. The inflammatory cells in the CLP + L-GA group and CLP + H-GA group decreased remarkably in comparison to the CLP group. Similarly, the semiquantitative score of histopathology in the CLP group was enormously higher compared to the Sham group (*p* < 0.01), and in the GA treatment rats (the CLP + L-GA group and CLP + H-GA group) that was notably lower than that in the CLP group (*p* < 0.01).

The apoptosis in the lung was observed by TUNEL (Figures 3(c) and 3(d)). The positive cell rate in the model rats (CLP group) was enormously larger in comparison to the Sham group (*p* < 0.01). Moreover, in the CLP + L-GA group and CLP + H-GA group, the values were both notably less compared to the CLP group (*p* < 0.01).

### 3.4. The Effects of GA on the Cell Number and Inflammatory Level in BACF of the Lung

**Figure 4:**
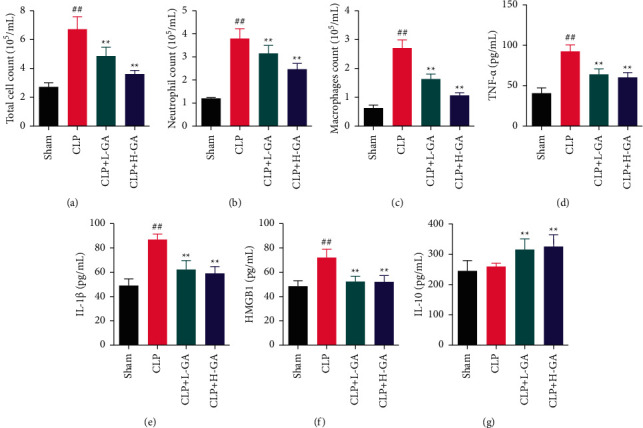
The numbers of inflammatory cell counts and levels of inflammatory cytokines in bronchoalveolar lavage fluid (BALF) of the lung ($\bar{x}$ ±*s*). (a–c) The counts of total cells, neutrophils, and macrophages in the BALF (*n* = 6). (d–f) The levels of pro-inflammatory cytokines (*n* = 6). (g) The levels of the anti-inflammatory cytokine (*n* = 6). CLP: cecal ligation and puncture. GA: glycyrrhizic acid, ^##^*p* < 0.01*vs.* the Sham group; ^*∗∗*^*p* < 0.01*vs.* the CLP group.

The counts of the total cell, neutrophil, and macrophages in the model rats (CLP group) were much greater in comparison to the Sham group (*p* < 0.01). Though, these cells were notably less in the GA treatment group compared to the CLP group (*p* < 0.01) (Figures 4(a)–4(c)).

The inflammatory level in BACF of the lung was tested by ELISA (Figures 4(d)–4(g)). The degrees of TNF-*α*, IL-1*β*, and HMGB1 in the model rats (CLP group) were tremendously superior to the Sham group (*p* < 0.01), and the IL-10 level was not significantly different (*p* > 0.05). Moreover, the degrees of TNF-*α*, IL-1*β*, and HMGB1 in the CLP + L-GA and CLP + H-GA groups were all notably lesser in comparison to the CLP group (*p* < 0.01). The IL-10 level in the CLP + L-GA and CLP + H-GA groups was both larger than in the CLP group (*p* < 0.01).

### 3.5. The Effects of GA on the Expression Degrees of HMGB1, TLR4, NF-*κ*B, P-38 MAPK, and ERK1/2 Proteins

**Figure 5:**
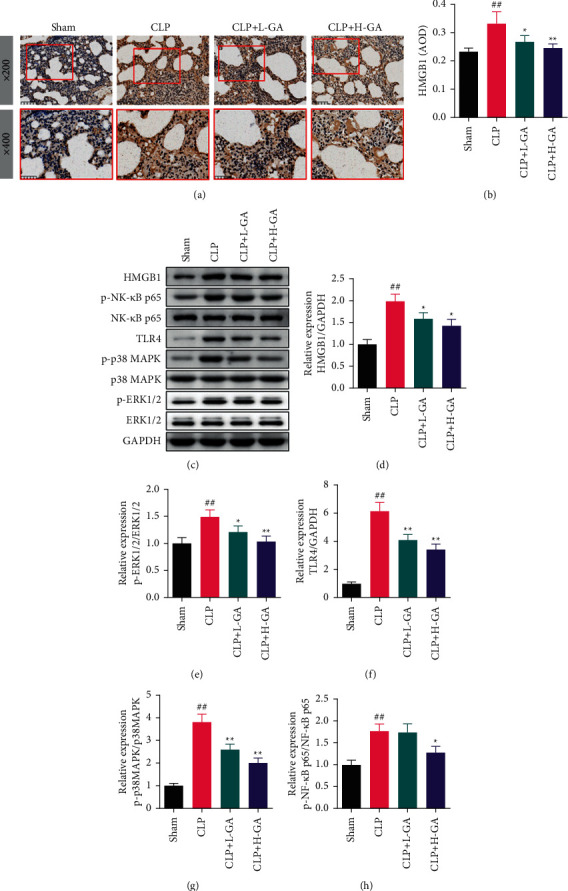
The expression degrees of HMGB1, TLR4, NF-*κ*B, p-38 MAPK, and ERK1/2 proteins ($\bar{x}$ ±*s*). (a, b) The level of HMGB1 protein was measured by immunohistochemistry (*n* = 6). (c–h) The expression degrees of HMGB1, TLR4, NF-*κ*B, p-38 MAPK, and ERK1/2 proteins analysis by Western blot (*n* = 3). GA: glycyrrhizic acid, CLP: cecal ligation and puncture, HMGB1: high mobility group box-1, TLR4: toll-like receptor4, NF-*κ*B: nuclear factor-*κ*B, p-38 MAPK: p-38 mitogen-activated protein kinase, ERK1/2: extracellular signal-regulated kinase l/2. ^##^*p* < 0.01*vs.* the Sham group; ^*∗*^*p* < 0.05, ^*∗∗*^*p* < 0.01*vs.* the CLP group.

According to the immunohistochemical results in Figures 5(a) and 5(b), it can be significantly observed that the protein expression of HMBG1 in the lung of the model rats (CLP group) was higher than that of the Sham group (*p* < 0.01). The protein expression of HMBG1 in the CLP + L-GA group and the CLP + H-GA group decreased considerably compared with the CLP group (*p* < 0.05 or *p* < 0.01).

The expression levels of proteins in the lung of CLP rats were measured by WB (Figures 5(c)–5(h)). The degrees of HMGB1 and TLR4 protein expressions in the CLP group were raised immensely compared to the rats as control (Sham group) (*p* < 0.01), and their expression degrees were reduced markedly in the CLP + L-GA group and the CLP + H-GA group (*p* < 0.05 or *p* < 0.01). The protein expression level of NF-*κ*B phosphorylation in the CLP group was increased very much than that in the rats as a control (Sham group) (*p* < 0.05), but it decreased remarkably in the CLP + H-GA yet (*p* < 0.01). Besides, the degrees of p-p-38 MAPK/p-38 MAPK and p-ERK1/2/ERK1/2 in the CLP group were tremendously superior to the Sham group, but their levels in the CLP + L-GA group and the CLP + H-GA group were lesser than those in the CLP group (*p* < 0.05 or *p* < 0.01).

## 4. Discussion

The mortality rate of patients with severe sepsis is about 26%, and globally, scientists estimate that 5.3 million people die of sepsis every year [22]. ALI/ARDS is one of the causes of high mortality of sepsis, its improvement went hand in hand with the levels of inflammation [23]. The study by Jiao et al. found that inhibiting inflammation improved sepsis-induced ALI [23]; The study by Wang et al. noticed that inhibited inflammation and improved ALI by inhibiting inflammatory corpuscles [24]. The improvement of inflammation was the key to the treatment of ARDS. GA has been shown to inhibit inflammation and apoptosis to improve ALI in sepsis rats [25]. Studies have found that GA could reduce IL-1*β*, MCP-1, COX2, HMGB1, and other inflammatory factors in lung tissues of mice with LSP-induced ALI [19]. Similarly, Gu et al. reported the inhibiting effect of GA on CLP-induced ARDS mice was related to the HMGB1/TLR9 pathway [21]. The results of this paper also proved that GA immensely improved the survival rate of CLP rats in a dose-dependent manner and effectively inhibited their systemic inflammatory response and the growth of bacteria in rat blood. An antibacterial study showed that the growth of Gram-positive *Staphylococcus aureus* could be completely inhibited by GA; besides, Zhang et al. found that chitosan, GA, and ZnO/palygorskite could supply a broad-spectrum antibacterial activity by the agar diffusion method [26, 27]. In short, GA had antibacterial and anti-inflammatory effects on experimental sepsis rats.

In addition, this paper analyzed the blood oxygen of CLP rats and observed the changes in HE-stained sections, MPO, and wet-to-dry ratio in the lung. It was found that 25 and 50 mg/kg enormously improved the abovementioned indexes. In particular, we found that 50 mg/kg GA had a similar inhibitory effect on inflammation as 25 mg/kg GA, but 50 mg/kg GA could better improve MPO, blood oxygen content, and dry-to-wet ratio in rats. It was proved that GA can significantly improve the lung function of CLP rats in a dose-dependent manner.

This paper observed that GA reduced the degrees of TNF-*α*, IL-*β*, and HMGB1 by ELISA. The levels of TNF-*α* and MCP-1 in plasma of 90 patients with severe sepsis were higher than those in the controls [28]. It was pointed out that improving inflammation can treat lung function and tissue injury. By analyzing the number of BACF cells, it was found that the number of neutrophils and macrophages in the BACF of CLP rats increased sharply. Neutrophil recruitment to the lung is a sign of ALI, which induces lung injury by releasing inflammatory mediators leading to pulmonary microcirculation block [29, 30]. Moreover, a study has proved that the exosomes of neutrophils induce M1 macrophage polarization and primes macrophage pyroptosis in ALI leading to injury [23]. In this study, GA administration reduced the number of neutrophils and macrophages in BACF, suggesting that its effect on improving ALI may be achieved by inhibiting neutrophils and macrophage activation.

HMGB1 is a multifunctional nuclear protein and affects a variety of inflammatory reactions by entering the cytoplasm, and scientists have noticed that its interaction with LPS can mediate caspase-11-dependent hepatitis in fatal sepsis [31]. HMGB1 has been shown to contribute to the pathogenesis of sepsis [32]. In recent years, it has been reported that inhibiting HMGB1 can improve inflammation and advance the prognosis of patients with sepsis [33]. In order to explore the function of GA on HMGB1, we analyzed the levels of the HMGB1 protein expression and its related proteins in the lung by IHC and WB. The results showed that HMGB1 and TLR4 decreased significantly, and the phosphorylation levels of NF-*κ*B, p-38 MAPK, and ERK1/2 were also significantly inhibited after injection of 50 mg/kg GA in GLP rat lung tissues. Studies have shown that HMGB1 can mediate the activation of NF-*κ*B and cause inflammation. Inhibiting HMGB1 acetylation can inhibit the NF-*κ*B pathway and improve the inflammatory response [34]. Gao's team demonstrated that HMGB1 can participate in the activation of p-38 MAPK through TLR4 [11]. In addition, it has been reported that HMGB1 promotes the activity of the p-38 MAPK and the ERK1/2 pathway to enhance the inflammatory response in human bronchial epithelial cells by combining RAGE which was consistent with the experimental results of this study [35]. Based on these foundations, it is indicated that the effect of GA on the activation of the NF-*κ*B, the p-38 MAPK, and the ERK1/2 pathways in CLP rats may be related to HMGB1. The detailed mechanism of GA regulating HMGB1 is worth exploring in subsequent research studies.

## 5. Conclusions

GA on the lung of septic rats improved survival, alleviated histopathological injury, inflammation, and cell apoptosis, and regulated the expression of HMGB1, p-NF-*κ*B, TLR4, p-p-38 MAPK, and ERK1/2 proteins, which were in a dose-dependent manner. In conclusion, the effect of GA on the treatment of ALI/ARDS induced by sepsis in rats may be achieved by inhibiting the activation of inflammation-related pathways by inhibiting HMGB1. This paper provides an experimental basis for the prevention and treatment of ALI/ARDS caused by sepsis.

## Data Availability

The data set used and analyzed in this study can be obtained from the corresponding author upon reasonable request.
